# Effect of Exercise on Breast Cancer: A Systematic Review and Meta-analysis of Animal Experiments

**DOI:** 10.3389/fmolb.2022.843810

**Published:** 2022-06-06

**Authors:** Yuxi Li, Xili Xiao, Yue Zhang, Wenjing Tang, Dongling Zhong, Tianyu Liu, Yuanyuan Zhu, Juan Li, Rongjiang Jin

**Affiliations:** ^1^ School of Health Preservation and Rehabilitation, Chengdu University of Traditional Chinese Medicine, Chengdu, China; ^2^ Department of Ophthalmology, Hospital of Chengdu University of Traditional Chinese Medicine, Chengdu, China; ^3^ School of Sports, Chengdu University of Traditional Chinese Medicine, Chengdu, China; ^4^ Affiliated Hospital of Panzhihua University Panzhihua, Panzhihua, China

**Keywords:** exercise, breast cancer, systematic review, meta-analysis, animal experiment

## Abstract

**Objective:** Exercise is reported to be beneficial for breast cancer. However, the results seem inconsistent. We conducted this systematic review and meta-analysis of animal experimental studies to fully understand the effect of exercise on breast cancer in animal model.

**Methods:** We searched databases from inception to April 2022 and manually searched related references to retrieve eligible studies. We screened eligible studies and extracted related data. We assessed the risk of bias and reporting quality using the SYstematic Review Centre for Laboratory animal Experimentation Risk of Bias tool and the Animal Research: Reporting of *In Vivo* Experiments guidelines 2.0, respectively. We summarized the study characteristics and findings of included studies and conducted meta-analysis with RevMan software. Subgroup analysis and sensitivity analysis were also performed.

**Results:** We identified 537 potential literatures and included 47 articles for analysis. According to the results of risk of bias assessment, only selective outcome reporting was in low risk of bias. Items of sequence generation, random outcome assessment, and incomplete outcome data were rated as high risk of bias. Most of other items were rated unclear risk of bias. In reporting quality assessment, all included articles reported grouping method and experimental procedures. However, no study provided information of the study protocol registration. Meta-analysis showed that, compared with sedentary lifestyle, exercise reduced more tumor weight (MD = −0.76, 95%CI −0.88 to −0.63, *p* = 0.85, *I* ^*2*^ = 0%) and tumor number per animal (MD = −0.61, 95%CI −0.91 to −0.31, *p* = 0.34, *I* ^*2*^ = 8%). Exercise decreased more tumor incidence than sedentary lifestyle both in motorized wheel/high-intensity (OR = 0.22, 95%CI 0.11 to 0.46, *p* = 0.09, *I* ^*2*^ = 41%) and free wheel/low-intensity treadmill running (OR = 0.45, 95%CI 0.14 to 1.44, *p* = 0.04, *I* ^*2*^ = 60%). Sensitivity analysis showed that the results were robust.

**Conclusion:** Exercise could reduce tumor weight, number of tumors per animal, and incidence of tumor in breast cancer model of mice and rats. However, the risk of bias items and reporting guidelines in preclinical studies should be concerned. Future research should consider standards of conducting and reporting preclinical studies and choose suitable exercise protocol for higher quality evidence of exercise for breast cancer.

## Introduction

Breast cancer is the main malignant tumor in females and is the leading cause of female cancer death worldwide (Fitzmaurice et al., 2019; Sharma, 2019). Globally, breast cancer has surpassed lung cancer as the most common cancer, with an estimated 2.3 million new cases and 6.9% mortality rate of them (Sung et al., 2021). Early detection, advanced treatment and an active lifestyle can improve breast cancer survival rates (Miller et al., 2019). The American Cancer Society recently issued guideline that recommended exercise for breast cancer prevention (Rock et al., 2020). Statistics also showed that, compared with inactivity, adults achieved 150–300 min of moderate-intensity exercise (or 75–150 min of vigorous-intensity exercise) per week could reduce 25–30% risk in breast cancer (Kushi et al., 2012). Currently, increasing studies have investigated whether exercise is beneficial for breast cancer during and after cancer treatment (Dieli-Conwright et al., 2018; Odynets et al., 2019; Rangel et al., 2019). However, the results are inconsistent and the relationship between exercise and breast cancer remains to be understood.

Experimental studies using animal models to mimic human disease can detail the onset, promotion, or progression of disease and identify the potential biological pathways (Hoffman-Goetz, 2003; Ashcraft et al., 2016), yet the current results of animal studies in exercise on tumor or the intensity of exercise effect were heterogeneous. Some studies found exercises were effective to slow tumor growth (Shalamzari et al., 2014; Alizadeh et al., 2018) and decrease tumor cell number of breast cancer (Alvarado et al., 2017), while other studies reported exercises did not inhibit tumor initiation (Steiner et al., 2013) or have no effect on the tumor volume (Garritson et al., 2019). The reason may be related with the different cancer phenotypes, the different model established methods of mammary adenoma and different exercise schemes (type, duration, intensity, and frequency of exercise).

To fully understand the effects of exercise on breast cancer in animal experiments, we retrieved preclinical studies focusing on the effect of exercise on breast cancer to comprehensively assess the risk of bias and reporting quality and conduct systematic review and meta-analysis.

## Methods

This meta-analysis was reported in accordance with the Preferred Reporting Items for Systematic Reviews and Meta-Analyses (PRISMA) statement (Page et al., 2021). The full PRISMA checklist is presented in Supplementary Table S1.

### Inclusion and Exclusion Criteria

The inclusion criteria were as follows: (Fitzmaurice et al., 2019) animal experiments investigating the effects of exercise on breast cancer; (Sharma, 2019) rats or mice model; (Sung et al., 2021) control group were set as sedentary control or other activity control; (Miller et al., 2019) outcomes included characteristics of tumor (including tumor volume, tumor weight, tumor number, tumor cell number, tumor incidence, and tumor growth rate); and (Rock et al., 2020) the language was limited to English and Chinese. The exclusion criteria included: (Fitzmaurice et al., 2019) studies with exercise combined diet, chemical therapy, or other therapies; (Sharma, 2019) duplicate studies; (Sung et al., 2021) studies reported outcomes of other tumors.

### Database and Search

We searched the literature from the following databases: PubMed, Embase, China National Knowledge Infrastructure (CNKI), Chinese Science and Technology Periodical Database (VIP), Wan Fang database and Chinese Biomedical Literature Database (CBM). We searched the literature from inception to 14 April 2022. Search terms combined breast cancer, exercise, and animal. The detailed search strategy is presented in Supplementary Table S2. We also searched websites, reference list from included articles and consulted experts to obtain possible eligible studies.

### Studies Selection

We used Endnote X9 to manage all records and identify duplicates. Two reviewers (YXL and YZ) independently screened titles and abstracts to select potential eligible studies according to inclusion criteria. Then, they read full texts of potential eligible studies to identify the final included literature. Any disagreement was resolved by consulting a third reviewer (RJJ).

### Data Extraction

Two reviewers (YXL and YZ) independently extracted data using an extraction table designed in advance. We extracted several literatures in advance and discussed the extraction results to ensure data consistency. We extracted the following data: 1) study characteristics; 2) methods of establishing animal model; 3) route of administration; 4) exercise design features; 5) tumor outcomes. Any disagreement was resolved through consensus or discussion with the third reviewer (JL).

### Risk of Bias Assessment

Two reviewers (XXL and DLZ) independently assessed the risk of bias using the SYstematic Review Centre for Laboratory animal Experimentation (SYRCLE)’s Risk of Bias (RoB) tool. The SYRCLE’s RoB tool specifically assesses the risk of bias in animal intervention studies (Hooijmans et al., 2014). The tool contains 10 entries in six aspects: selection bias (items 1–3), performance bias (items 4–5), detection bias (items 6–7), attrition bias (items 8), reporting bias (items 9) and other biases (items 10). Each item is rated as “yes” (low risk of bias), “no” (high risk of bias) and “unclear” (if insufficient details are obtained). Any disagreement was resolved through discussion or by consulting a third reviewer (YYZ).

### Reporting Quality Assessment

Two reviewers (XXL and DLZ) independently assessed reporting quality using the Animal Research: Reporting of *In Vivo* Experiments (ARRIVE) guidelines 2.0, respectively. The ARRIVE 2.0 consists of 21 items, which divided the items into 2 sets, the “ARRIVE Essential 10” which constitutes the minimum requirement, and the “Recommended Set,” which describes the research context. “ARRIVE Essential 10” contains detailed information on the study design, the sample size, measures to reduce subjective bias, outcome measures, statistical methods, the animals, experimental procedures, and results. “Recommended Set” includes detailed information on the abstract, background, objectives, ethical statement, housing and husbandry, animal care and monitoring, interpretation/scientific implications, generalisability/translation, protocol registration, data access, and declaration of interests. Each item is judged as “Yes”, “No”, and “Partial Yes”. Any disagreement was resolved through discussion or by consulting a third reviewer (YYZ).

### Data Analysis

We summarized the study characteristic and findings of included literatures and presented the results in tables. We used diagram and tables to summarize the results of risk of bias and reporting quality assessment. RevMan software (version 5.3.5) was utilized to conduct the data analysis. The MD (Mean difference) was utilized for data measurement of continuous outcomes, and OR (Odds ratio) was for dichotomous variable. All of them were expressed with a 95% confidence interval (CI). We assessed the heterogeneity between studies using Cochrane’s *Q* test and *I* ^*2*^ test. We conducted sensitivity analysis using Stata/SE 15.1 software to explore the robustness of results. We also performed subgroup analysis according to intensity of exercise. Funnel plot was used to evaluate publication bias. *p* < 0.05 was considered significant. For those outcomes with high heterogeneity, we conducted descriptive analysis.

## Results

**FIGURE 1:**
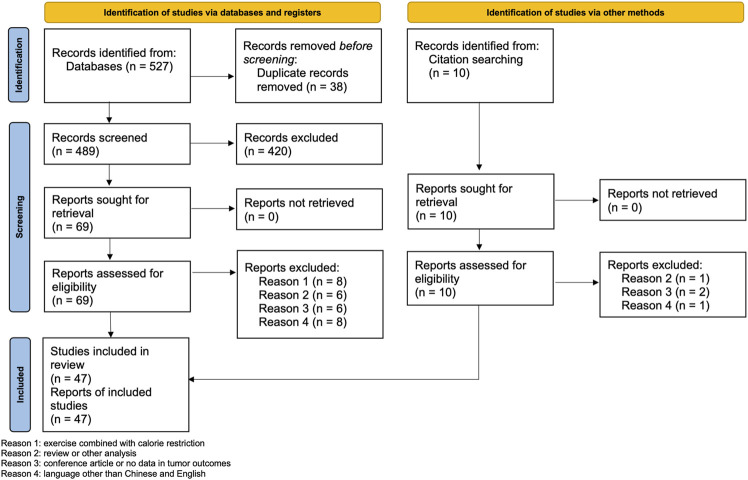
PRISMA 2020 flow diagram.

We identified 537 potential literatures. After removal of duplicates and initial screening, we excluded 420 articles, and downloaded 79 full texts for secondary screening. Finally, we included 47 articles for analysis. The excluded studies and the reasons for exclusion are listed in Supplementary Table S3. Figure 1 shows the flow chart of the selection process.

### Study Characteristics

The characteristics of studies are presented in Supplementary Table S1. The methods of establishing tumor models include: carcinogenic agent 1- methyl-1-nitrosourea (MNU) injection (n = 15, 31.9%), 4T1 breast tumor cells injection (n = 8, 17.0%), 7, 12-dimethylbenz(a)anthracene (DMBA) oral or injection (n = 6, 12.8%), MC4-L2 cells injection (n = 6, 12.8%), MDA-MB-231 breast carcinoma cells implantation (n = 3, 6.4%), EO771 breast or B16-F10 melanoma tumor cells inoculation (n = 2, 4.3%), transgenic mice (n = 1, 2.1%), BCAP-37 breast cancer cells inoculation (n = 1, 2.1%), and breast adenocarcinoma cells inoculation without mention of tumor cell lines (n = 1, 2.1%), and transgenic mice with spontaneous breast cancer (n = 1, 2.2%). Types of exercise are as follows: treadmill/wheel running (n = 43, 91.5%), including voluntary and motorized running, swim training (n = 3, 6.4%), and interval aerobic training (n = 1, 2.1%). The duration of exercise ranges from 2 to 36 weeks. Control groups include tumor/non-trained, sedentary, locked running wheels, immobile treadmill and shallow water pool. The animal sample sizes varies from 12 to 150.

### Risk of Bias Assessment

**FIGURE 2:**
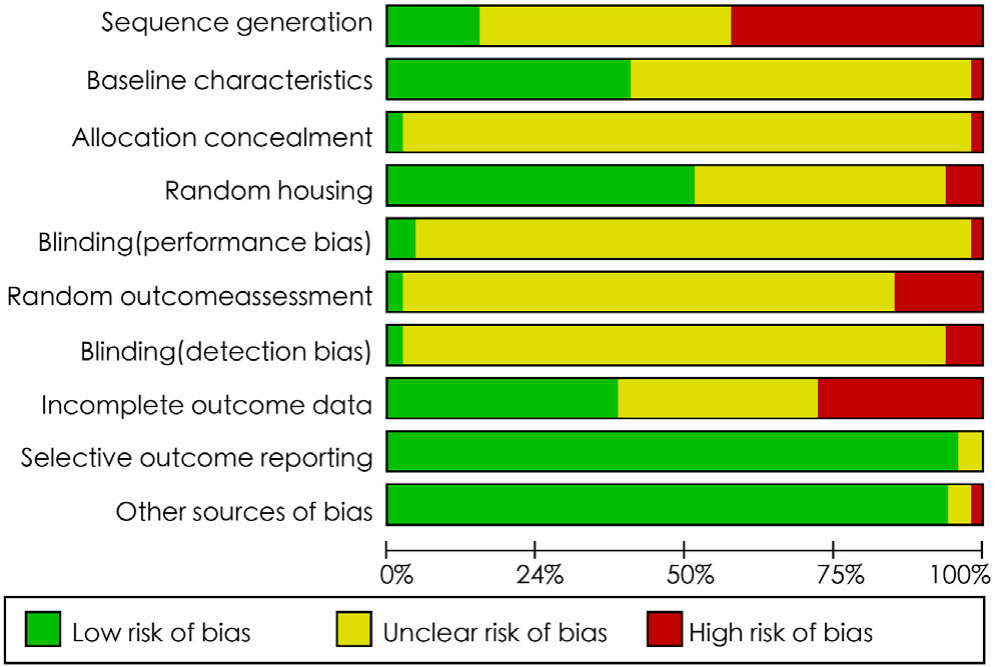
SYRCLE’s RoB tool for assessing risk of bias.

Figure 2 shows the results of the risk of bias assessment. Item 9 “selective outcome reporting” and item 10 “other sources of bias” presented low risk of bias, with rate of 95.7 and 93.6%, respectively. Item 1 “sequence generation”, item 6 “random outcome assessment”, and item 8 “incomplete outcome data” were rated with high risk of bias, with rate of 42.6, 14.9 and 27.7%, respectively. Item 2 “baseline characteristics” and item 4 “random housing” showed unclear and low risk of bias, respectively. In the remaining items, unclear risk of bias was observed in most articles.

### Reporting Quality Assessment

As shown in Table 1, all studies reported the most adequate information in grouping method in study design section (1a) and what and how was done in experimental procedures (9a), with a frequency of 100%. Details of experimental animals (8a), when and how often the experimental was done (9b), descriptive statistics for each experimental group (10a) were reported in 44 (95.7%) studies. Definition of outcome measures (6a), statistical methods (7a) and background of the study (12a) were reported in 43 (93.5%) studies. None of the 47 studies provided information on the study protocol registration.

**TABLE 1:** Reporting rate of ARRIVE guidelines 2.0 for included studies.

Domain/Number		Item	Reported (Number, %)		
		The ARRIVE Essential 10	Y	N	NA
Study design	1a	The groups being compared, including control groups. If no control group has been used, the rationale should be stated	100		
	1b	The experimental unit (e.g., a single animal, litter, or cage of animals)	89.4	8.5	2.1
Sample size	2a	Specify the exact number of experimental units allocated to each group, and the total number in each experiment. Also indicate the total number of animals used	68.1	29.8	2.1
	2b	Explain how the sample size was decided. Provide details of any a priori sample size calculation, if done	2.1	93.6	4.3
Inclusion and exclusion criteria	3a	Describe any criteria used for including or excluding animals (or experimental units) during the experiment, and data points during the analysis. Specify if these criteria were established a priori. If no criteria were set, state this explicitly	21.3	76.6	2.12
	3b	For each experimental group, report any animals, experimental units, or data points not included in the analysis and explain why. If there were no exclusions, state so	19.1	78.7	2.1
	3c	For each analysis, report the exact value of n in each experimental group	38.3	57.4	4.3
Randomisation	4a	State whether randomisation was used to allocate experimental units to control and treatment groups. If done, provide the method used to generate the randomisation sequence	17	74.5	8.5
	4b	Describe the strategy used to minimise potential confounders such as the order of treatments and measurements, or animal/cage location. If confounders were not controlled, state this explicitly	6.4	93.6	
Blinding	5	Describe who was aware of the group allocation at the different stages of the experiment (during the allocation, the conduct of the experiment, the outcome assessment, and the data analysis)	2.1	95.7	2.1
Outcome measures	6a	Clearly define all outcome measures assessed (e.g., cell death, molecular markers, or behavioural changes)	93.6	4.3	2.1
	6b	For hypothesis-testing studies, specify the primary outcome measure, i.e., the outcome measure that was used to determine the sample size	74.5	21.3	4.3
Statistical methods	7a	Provide details of the statistical methods used for each analysis, including software used	93.6	6.4	
	7b	Describe any methods used to assess whether the data met the assumptions of the statistical approach, and what was done if the assumptions were not met.	23.4	74.5	2.1
Experimental animals	8a	Provide species-appropriate details of the animals used, including species, strain and substrain, sex, age or developmental stage, and, if relevant, weight	95.7	2.1	2.1
	8b	Provide further relevant information on the provenance of animals, health/immune status, genetic modification status, genotype, and any previous procedures	70.2	27.7	2.1
Experimental procedures	9a	What was done, how it was done, and what was used	100		
	9b	When and how often	95.7	4.3	
	9c	Where (including detail of any acclimatization periods)	76.6	23.4	
	9d	Why (provide rationale for procedures)	17	83	
Results	10a	Summary/descriptive statistics for each experimental group, with a measure of variability where applicable (e.g., mean and SD, or median and range)	95.7	4.3	
	10b	If applicable, the effect size with a confidence interval	19.1	38.3	42.6
**The recommended set**					
Abstract	11	Provide an accurate summary of the research objectives, animal species, strain and sex, key methods, principal findings, and study conclusions	91.5	8.5	
Background	12a	Include sufficient scientific background to understand the rationale and context for the study and explain the experimental approach	93.6	6.4	
	12b	Explain how the animal species and model used address the scientific objectives and, where appropriate, the relevance to human biology	21.3	66	12.8
Objectives	13	Clearly describe the research question, research objectives and, where appropriate, specific hypotheses being tested	89.4	4.3	6.4
Ethical statement	14	Provide the name of the ethical review committee or equivalent that has approved the use of animals in this study and any relevant license or protocol numbers (if applicable). If ethical approval was not sought or granted, provide a justification	70.2	27.7	2.1
Housing and husbandry	15	Provide details of housing and husbandry conditions, including any environmental enrichment	76.6	21.3	2.1
Animal care and monitoring	16a	Describe any interventions or steps taken in the experimental protocols to reduce pain, suffering, and distress	34	63.8	2.1
	16b	Report any expected or unexpected adverse events	12.8	87.2	
	16c	Describe the humane endpoints established for the study, the signs that were monitored, and the frequency of monitoring. If the study did not set humane endpoints, state this	40.4	55.3	4.3
Interpretation/scientific implications	17a	Interpret the results, taking into account the study objectives and hypotheses, current theory, and other relevant studies in the literature	89.4	10.6	
	17b	Comment on the study limitations, including potential sources of bias, limitations of the animal model, and imprecision associated with the results	17	83	
Generalisability/translation	18	Comment on whether, and how, the findings of this study are likely to generalize to other species or experimental conditions, including any relevance to human biology (where appropriate)	21.3	74.5	4.3
Protocol registration	19	Provide a statement indicating whether a protocol (including the research question, key design features, and analysis plan) was prepared before the study, and if and where this protocol was registered		100	
Data access	20	Provide a statement describing if and where study data are available	10.6	89.4	
Declaration of interests	21a	Declare any potential conflicts of interest, including financial and nonfinancial. If none exist, this should be stated	31.9	68.1	
	21b	List all funding sources (including grant identifier) and the role of the funder(s) in the design, analysis, and reporting of the study	40.4	59.6	

AbbreviationsY = yes; N = no; NA, not available.

### Meta-Analysis

#### Tumor Weight

**FIGURE 3:**
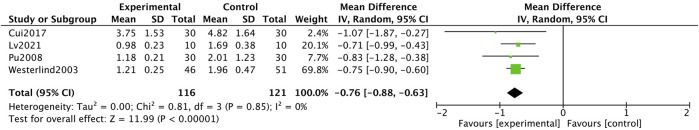
Forest plot of the influence of exercise vs sedentary control on tumor weight.

We included 7 studies for meta-analysis, which showed tumor weight in exercise group reduced more than control group (MD = −0.52, 95%CI −0.91 to −0.12, *p* < 0.00001, *I* ^*2*^ = 86%). By exploring heterogeneity, we found the duration of exercise of Faustino 2016 (Faustino-Rocha et al., 2016) and Faustino 2017 (Faustino-Rocha et al., 2017) were 35 weeks, Woods 1994 (Woods et al., 1994) was 2 weeks, while exercise time in other studies ranged from 4 to 16 weeks. After removing these 3 studies, the *I* ^*2*^ dropped to 0%, which indicated there was no heterogeneity, and the result remained the same (MD = −0.76, 95%CI −0.88 to −0.63, *p* < 0.00001, *I* ^*2*^ = 0%) (Figure 3).

#### Tumor Number

**FIGURE 4:**
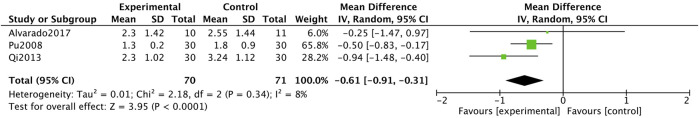
Forest plot of the influence of exercise vs sedentary control on tumor number.

Pooled data from 3 studies revealed that wheel running decreased the number of tumors per animal (MD = −0.61, 95%CI −0.91 to −0.31, *p* < 0.0001, *I* ^*2*^ = 8%) (Figure 4). Murphy 2011 (Murphy et al., 2011), Thompson 2010 (Thompson et al., 2010) and Zhu 2008 (Zhu et al., 2008) also reported running decreased the number of tumors in animal. However, when we synthesized the data, we found that the heterogeneity was too high after analyzing their study characteristics, we found the animal model and exercise protocol may be the reasons of heterogeneity.

#### Tumor Incidence

**FIGURE 5:**
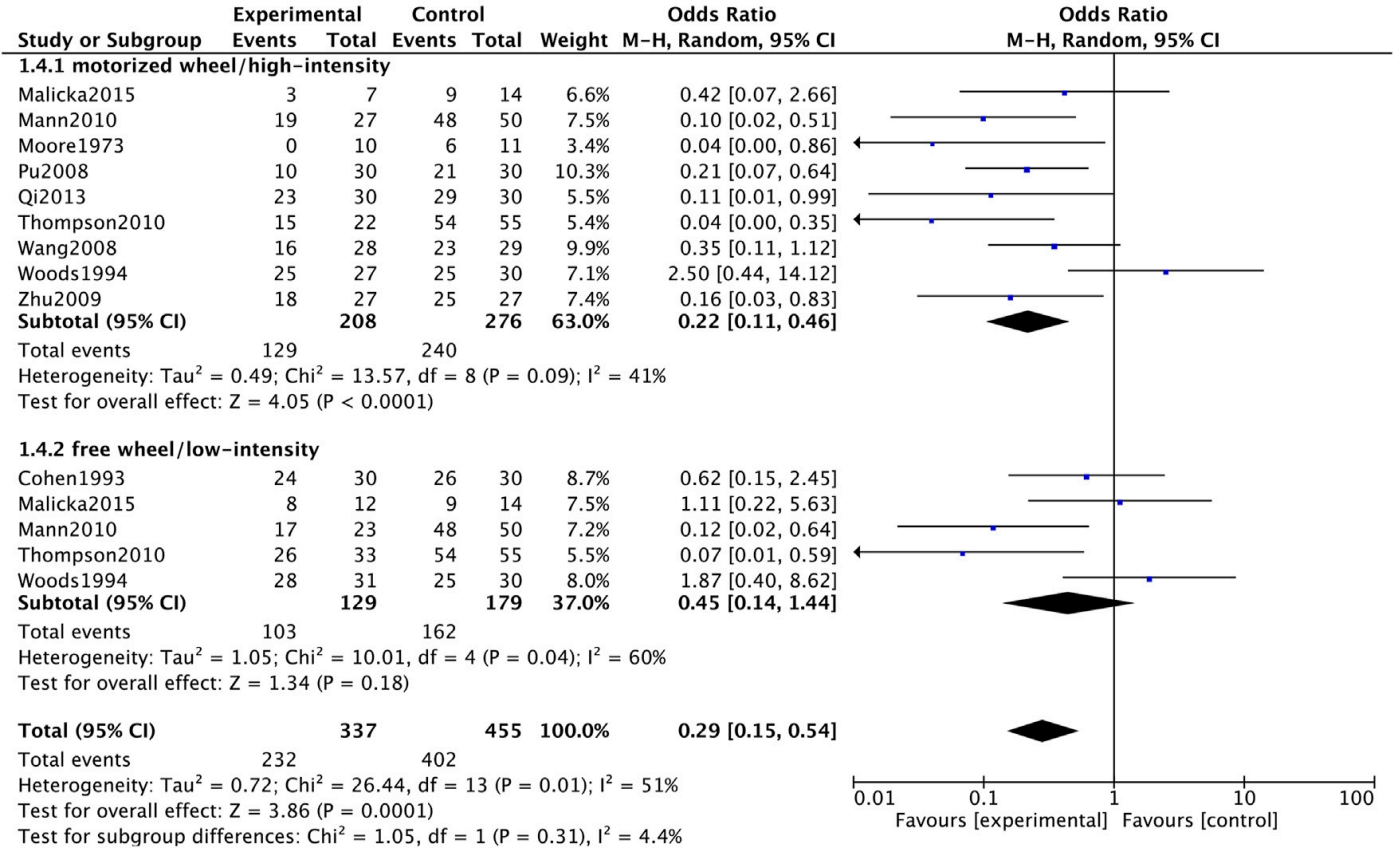
Forest plot of the influence of exercise vs control on tumor incidence.

**FIGURE 6:**
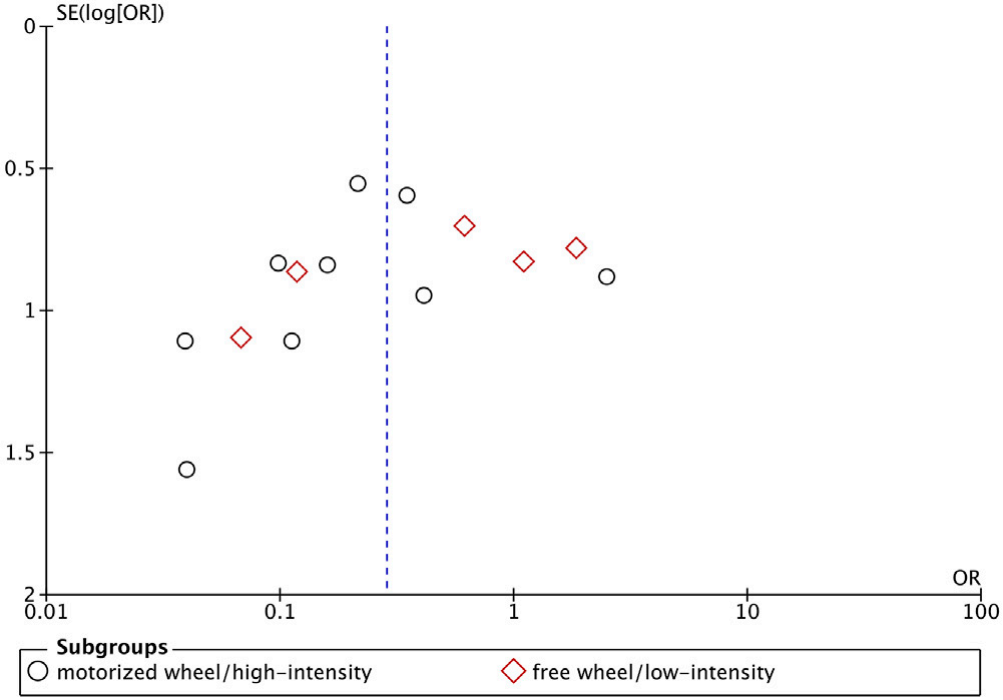
Funnel plot of the influence of exercise vs control on tumor incidence.

As for tumor incidence, we separated data based on different intensity of exercise to conduct subgroup meta-analysis. Based on the exercise protocol of original studies, we grouped motorized wheel and high-intensity treadmill running together, while free wheel and low-intensity treadmill running studies were grouped in another group. Compared with sedentary group, both motorized wheel/high-intensity (OR = 0.22, 95%CI 0.11 to 0.46, *p* < 0.0001, *I* ^*2*^ = 41%) and free wheel/low-intensity treadmill running (OR = 0.45, 95%CI 0.14 to 1.44, *p* = 0.18, *I* ^*2*^ = 60%) could decrease tumor incidence (Figure 5). The asymmetric funnel plot showed publication bias might exist (Figure 6).

### Sensitivity Analysis

We performed sensitivity analysis for outcomes of meta-analysis to test the robustness of the results. We found the result of each study did not have any important impacts on the overall findings (Supplementary Table S4).

### Descriptive Analysis

Among the included studies, 21 studies (Steiner et al., 2013; Shalamzari et al., 2014; Alvarado et al., 2017; Alizadeh et al., 2018; Murphy et al., 2011; Aveseh et al., 2015; Leila et al., 2015; Malicka et al., 2015; Isanejad et al., 2016; Cui, 2017; Nasiri et al., 2017; Lyv Di et al., 2021; Qi et al., 2013; Siewierska et al., 2018; Siewierska et al., 2020; Wen et al., 2010; Gholamian et al., 2020; Vulczak et al., 2020; Wennerberg et al., 2020) reported that exercise could decrease the tumor volume, 1 (Faustino-Rocha et al., 2016) reported negative effects of exercise, and 2 (Smeda et al., 2017; Garritson et al., 2019) reported that exercise had no effect on the tumor volume.

3 studies (Cohen et al., 1993; Thompson et al., 1995; Zhu et al., 2008) reported that exercise reduced the tumor multiplicity, while one study (Colbert et al., 2009) found multiplicity of mammary carcinomas increased in wheel running animals.

8 studies (Jones et al., 1985; Welsch et al., 1995; Westerlind et al., 2003; Steiner et al., 2013; Shalamzari et al., 2014; Bianco et al., 2017; Nasiri et al., 2017; Vulczak et al., 2020) reported exercise delayed tumor growth rate, while Buss (Buss et al., 2020) and da Costa (da Costa et al., 2021) found exercise did not affect tumor growth rate.

1 study (Alvarado et al., 2017) reported that exercised mice did not develop any metastasis, while 2 pulmonary metastases were observed in the sedentary group. In Goh’s study (Goh et al., 2013), no difference in tumor growth was observed between runners and non-runners.

As for other outcomes: 3 studies (Cohen et al., 1993; Pu et al., 2008; Faustino-Rocha et al., 2016) reported exercise increased the tumor latency; 2 studies (Jones et al., 1985; Faustino-Rocha et al., 2016) reported exercise training could enhance vascularization.

## Discussion

The results of this study indicated that exercise could reduce tumor weight, number of tumors per animal, and incidence of tumor in breast cancer model of mice and rats. However, we found most of included studies failed to report some items in ARRIVE guideline, such as sample size calculation, randomization, blinding methods, which also led to unclear risk of bias in SYRCLE assessment.

Our study found that exercise reduced tumor incidence. In contrast, Cohen (Cohen et al., 1993) reported no effect on overall tumor incidence in exercise group animals, Woods (Woods et al., 1994) and Colbert (Colbert et al., 2009) found exercise might increase tumor incidence. Different animal models and exercise protocols may be the reasons for inconsistent findings.

Our study showed exercise could decrease tumor weight. Although, the results in tumor volume were too heterogeneous to be synthesized, 21 studies reported beneficial effects of exercise in tumor volume. Smeda’s study (Smeda et al., 2017) demonstrated that spontaneous voluntary wheel running had no effect on the volume and size of primary breast tumor. In Faustino’s research (Faustino-Rocha et al., 2016), the tumors’ weight and volume were higher in exercised animals compared with sedentary ones. The author explained that this might be related with the enhancement of blood perfusion.

Significant reduction in tumor number in exercised animals was noted in our study. Among included studies, Steiner’s study showed that voluntary wheel running was associated with an increased number of tumors developing in mice. This negative effect of exercise in tumor number may attribute to the different animal model they used. The model in Steiner’s study was the representative, triple-negative C3 (1)/SV40Tag transgenic mouse model. The author interpreted that voluntary exercise might not overcome the highly tumorigenic phenotype induced by the inactivation of two primary tumor suppressors, p53 and pRb (Green et al., 2000).

Malicka (Malicka et al., 2015) reported tumor incidence increased in low intensity exercise group, while dropped in moderate and high intensity exercise group. Our meta-analysis also came up with the same results, except for the result about low intensity exercise on tumor incidence due to limited research data. A previous review pointed out that as exercise intensity increased, it is more likely that physical activity would inhibit carcinogenesis (Thompson, 1994). The present results also demonstrated that different exercise protocols may be associated with different influences on tumor outcomes. Negative effects were more likely to be found in voluntary or low-intensity exercises, whilst forced or moderate, high-intensity exercises studies appeared to have better results. However, previous correlation analysis revealed that benefits were associated with low-intensity exercise, and voluntary exercise appeared to have more positive influence on the incidence, multiplicity and weight of tumors than forced exercise (Figueira et al., 2018).

Our results showed that most of included studies were assessed as unclear risk of bias, and they rarely followed the reporting guidelines. In 2002, the Lancet published an influential commentary (Sandercock and Roberts, 2002) mentioned the importance of risk of bias of animal studies. Since then, the awareness of risk of bias of preclinical studies had been increasing. Several practical guidelines were issued to facilitate well-informed decision-making evidence from animal studies (Hooijmans et al., 2012; Vesterinen et al., 2014; Soliman et al., 2020). The implementation of risk of bias tool and reporting guideline will enhance reliability and robustness of evidence from animal studies. More than that, this may subsequently improve the transformation of preclinical results into clinical experiments. In the field of exercise-oncology research, there are several risk of bias items and reporting issues should be concerned: (Fitzmaurice et al., 2019) state criteria for including or excluding animals; (Sharma, 2019) describe randomization and allocation of animals; (Sung et al., 2021) describe blinding methods of each stage of experiment; (Miller et al., 2019) report the implantation methods of tumor cell lines or induction of orthotopic tumors; (Rock et al., 2020) mention protocol of exercise intervention (forced or voluntary, exercise intensity, duration, frequency); (Kushi et al., 2012) design and report a prior-registered study protocol.

### Limitations

There are few limitations in our study. First, we were unable to do meta-analysis for some of the findings, as the heterogeneity among the included studies allowed us to only summarize and describe their results. Second, unclear risk of bias and relatively high risk of bias may affect the results. Third, we only included studies published in Chinese and English, which language bias was inevitable.

## Conclusion

Exercise could reduce tumor weight, number of tumors per animal, and incidence of tumor in breast cancer model of mice and rats. However, the risk of bias items and reporting issues in preclinical studies should be concerned. Future research should consider standards of conducting and reporting preclinical studies and choose suitable exercise protocol for higher quality evidence in exercise for breast cancer.
