# Supplementary material for: Effect of Exercise on Breast Cancer: A Systematic Review and Meta-analysis of Animal Experiments
Source: Front Mol Biosci. 2022 Jun 6;9:843810. doi: 10.3389/fmolb.2022.843810 (PMC9208379; doi:10.3389/fmolb.2022.843810)
Supplement: Supplementary file 1 [file Table1.docx]

**Supplementary Table 1 Characteristics of included studies**

| **Author, year** | **Animal model** | **Route of administration** | **Exercise protocol** | | | **Control method** | **Sample size** | **Tumor outcomes** |
| --- | --- | --- | --- | --- | --- | --- | --- | --- |
|  |  |  | **Type** | **Duration** | **Frequency** | **Type** |  |  |
| Abdalla2013 | Female Balb/c mice (8 weeks old) | An oral administration of 7, 12-dimethylbenz(a)anthracene (DMBA) at a concentration of 1mg/ml by daily gavage for 6 weeks. | Swim training | 8 weeks | 5 days/week | Sedentary control | 56 | ↓Tumor incidence in the exercise group animals |
| Alizadeh2018 | Female Balb/c mice (6-8 weeks old) | MC4-L2 mammary adenocarcinoma tumors were cut into pieces of than 0.2-0.3 cm3, and subcutaneously transplanted into the animals' right flank. | Treadmill running | 6 weeks | 5 days/week | Sedentary control | 16 | ↓Tumor volume in the exercise group animals;↓Tumor weight in the exercise group animals |
| Alvarado2017 | Female Sprague-Dawley rats | An intraperitoneal injection (50 mg/kg body weight) of the carcinogenic agent 1- methyl-1-nitrosourea (MNU) | Treadmill running | 35 weeks | 5 days/week | Sedentary control | 50 | ↓Tumor volume in the exercise group animals;  ↓Tumor cell number in the exercise group animals;  ↓Tumor metastasis in the exercise group animals |
| Amani2014 | Female Balb/c mice (3-5 weeks old) | One million MC4-L2 cells were injected subcutaneously into the right up thigh of the mice. | Treadmill running | 6 weeks | 5 days/week | Sedentary control | 40 | ↓Tumor volume in the exercise group animals;↓Tumor growth rate in the exercise group animals |
| Aveseh2015 | Female Balb/c mice (5 weeks old) | MC4-L2 cells (1.2 × 106 cells in 100 μl of PBS) were injected subcutaneously into the right dorsal mammary fat pad. | Treadmill running | 7 weeks | Every day | Sedentary control | 50 | ↓Tumor volume in the exercise group animals; ↓Tumor weight/body weight in the exercise group animals |
| Bianco2017 | Female Balb/c mice (8 weeks old) | 4T1 mouse breast tumor cells (2x105) were injected into the mammary gland of host mice. | Swim training | 4 weeks | 5 days/week | Sedentary control | 40 | ↓Tumor growth in the exercise group animals; ↓Tumor incidence in the exercise group animals |
| Buss2018 | Female ApoE(-/-) mice (6–10weeks old) | Mice were injected into the 4th mammary fat pad with 2*105 EO771 murine medullary breast adenocarcinoma cells. | Voluntary treadmill running | NR | NR | Sedentary control | 39 | Wheel running appeared to reduce internal metastases, but did not affect T cell infiltrate or the proportion of regulatory and cytotoxic T cells within the tumor; wheel running can slow the establishment of primary and secondary EO771 breast tumors and induce beneficial changes in the breast tumor microenvironment in ApoE |
| Buss2020 | Female C57BL/6 mice | Mice were inoculated with EO771 breast or B16-F10 melanoma tumor cells | Voluntary treadmill running | NR | NR | Sedentary control | 24 | ↑Heart to body weight ratio in the exercise group animals; Exercise did not affect vascularity, perfusion, hypoxia or tumor growth rate in either tumor type; EO771 tumors had a more aggressive phenotype than B16-F10 tumors |
| Cohen1993 | Female Seprague-Dawley rats (8 weeks old) | Administered by intragastric instillation of a single dose (5 or 10 mg/kg body weight) of DMBA. | Voluntary treadmill running | 19 weeks | NR | Sedentary control | 120 | ↓Total tumor numbers in exercise group animals; ↑Tumor latency in the low but not the high DMBA active groups;  ↓Tumor multiplicity in the high, but not the low DMBA exercised group; No effect on overall tumor incidence in exercise group animals. |
| Colbert2009 | Female heterozygous p53-deficient (p53+/−): MMTV-Wnt-1 transgenic mice | / | Treadmill running | (Mice were sacrificed when tumors reached 1.5 cm) | 5 days/week | Sedentary control | 63 | ↑the rate of tumor development in treadmill running animals; ↓survival time in treadmill running animals; ↑ the proportion of mice with multiple mammary carcinomas in treadmill running animals.  ↑ incidence and multiplicity of mammary carcinomas in wheel running animals |
| Cui2017 | Female Sprague-Dawley rats | 0.2 mL of 4T1 cell suspension was inoculated subcutaneously in the right axillary of rats. | Treadmill running | 8 weeks | 6 days/week | Sedentary control | 60 | ↓Tumor volume in exercise group animals;  ↓Tumor weight in exercise group animals |
| da Costa2021 | Female mice carrying the polyomavirus middle T antigen (mouse mammary tumor virus [MMTV]‐PyMT+), a transgenic mouse model of spontaneous breast cancer with C57BL6/J genetic background | Transgenic mice with spontaneous breast cancer | Treadmill running | NR | 5 days/week | Sedentary control | 22 | Tumor growth rate was not significantly different between sedentary and exercise group animals. |
| Faustino201 | Female Sprague-Dawley rats (7 weeks old) | A single intraperitoneal administration of the carcinogen agent MNU at a dose of 50 mg/kg | Treadmill running | 35 weeks | 5 days/week | Sedentary control | 50 | Exercised animals showed less tumors with an increased latency period;  ↑Vascularization of tumors in exercise group animals; ↑Tumor volume in exercise group animals; ↓Tumor number in exercise group animals |
| Faustino2017 | Female Sprague–Dawley rats (4–5 weeks old) | Intraperitoneal injection of MNU at a dose of 50 mg/kg. | Treadmill running | 35 weeks | 5 days/week | Sedentary control | 20 | ↓The number of malignant lesions in the exercise group animals; ↓The number of mammary tumors and lesions in the exercise group animals |
| Gholamian2020 | Female Balb/c mice (3-5 weeks old) | One million cells from 4T-1 cell line were cultured and subcutaneously injected into the upper part of the thighs. | Treadmill running | 4 weeks | 5 days/week | Sedentary control | 32 | ↓Tumor volume in the exercise group animals |
| Goh2013 | F1 hybrid female mice (6 weeks old) | / | Voluntary treadmill running | 10 weeks | NR | Sedentary control | 25 | ↓Tumor sizes in the exercise group animals; No differences in tumor burden or metastatic burden were observed between runners and non-runners. |
| Isanejad2016 | Female Balb/c mice (6-8 weeks old) | MC4-L2 cells (1×106 cells in a final volume of 0.1 ml) were inoculated in the right inguinal flank near to back of animals. | Treadmill running | 5 weeks | 5 days/week | Sedentary control | 64 | ↓Tumor volume(size) in the exercise group animals |
| Jacob2019 | Female mice | One million 4T1 mammary carcinoma cells were inoculated in the mammary fat pad. | Treadmill running | 4 weeks | NR | Sedentary control | NR | No change in tumor volumes or tumor weights in the exercise group animals; ↓The proportions of tumor-infiltrating MDScs in the exercise group animals |
| Jones2005 | Female athymic nu/nu mice (3-4 weeks old) | MDA-MB-231 breast carcinoma cells were implanted into the right flank of female mice. | Treadmill running | 8 weeks | 5 days/week | Sedentary control | 42 | ↑Survival rate in the exercise group animals. |
| Jones2010 | Athymic homozygous female mice (3–4 weeks old) | MDA-MB- 231 were injected orthotopically into the right dorsal mammary fat pad. | Voluntary treadmill running | NR | NR | Sedentary control | 50 | ↓Tumor growth (survival) in the exercise group animals;  Tumors from exercising animals had significantly improved blood perfusion/vascularization |
| Leila2015 | Female Balb/c mice (4-5 weeks old) | One million MC4-L2 estrogen-receptor-positive breast-cancer cells were injected subcutaneously into each mouse’s right upper thigh. | Treadmill running | 6 weeks | 5 days/week | Sedentary control | 20 | ↓Tumor volume in the exercise group animals |
| Lv2021 | Female Balb/c mice (3-5 weeks old) | Subcutaneously injected with 4T1 cellular suspension of breast adenocarcinoma (0.2 mL) under the right armpit. | Treadmill running | 4 weeks | 5 days/week | Sedentary control | 20 | ↓Tumor volume in the exercise group animals;↓Tumor weight in the exercise group animals |
| Mafalda2018 | Female Sprague-Dawley rats (38 days old) | Injected intraperitoneally with MNU given in a dose of 50 mg/kg. | Treadmill running | 36 weeks | 6 days/week | Sedentary control | 50 | ↓Malignancy incidence in the exercise group animals; ↓Tumor aggressiveness in the exercise group animals |
| Malicka2015 | Female Sprague–Dawley rats (4 weeks old) | Intraperitoneally injected with 180 mg/kg body weight of MNU. | Three-position treadmill (speed of the treadmill and the duration of exercise were gradually increased depending on the group’s established level of intensity) | 12 weeks | 5 days/week | Sedentary control | 50 | ↑Tumor incidence in low intensity exercise group animals;  ↓Tumor incidence in moderate and high intensity exercise group animals;  ↓Tumor volume in the exercise group animals; ↑TUNEL-positive cancer cells may in the exercise group animals |
| Mann2010 | Female Sprague-Dawley rats (20 days) | Intraperitoneally injected with 50 mg/kg body weight of MNU. | Treadmill running | NR | NR | Sedentary control | 100 | ↓Tumor number in exercise group animals; ↓Tumor incidence in exercise group animals; ↓Induced citrate synthase activity in exercise group animals |
| Moore1973 | Sprague-Dawley rats (50 days old) | DMBA Intravenous administration | Treadmill running | 19 weeks | 7 days/week | Sedentary control | 21 | ↓Tumor incidence in exercise group animals |
| Murphy2011 | Female C3(1)SV40Tag mice | NR | Treadmill running | 20 weeks | 6 days/week | Sedentary control | 26 | ↓Tumor volume in the exercise group animals; ↓Tumor number in the exercise group animals. |
| Nasiri2017 | Female Balb/c mice | One million suspended MC4-L2 cells in PBS buffer were subcutaneously injected into the right side of the mice. | Treadmill running | 10 weeks | 5 days/week | Sedentary control | 12 | ↓Tumor volume in the exercise group animals;  ↓Tumor growth in the exercise group animals |
| Pu2008 | Female Sprague-Dawley rats (4 weeks old) | Intraperitoneal injection of 25mg/kg body weight of MNU. | Treadmill running | 16 weeks | NR | Sedentary control | 60 | ↓Tumor incidence in exercise group animals; ↓Tumor number in exercise group animals; ↓Tumor weight in exercise group animals; ↑Tumor latency in exercise group animals |
| Qi2013 | Female SPF Sprague-Dawley rats (7 weeks old) | DMBA sesame oil solution once (10 mg/mL). | Treadmill running | 18 weeks | 4 days/week (first 2 weeks), 5 days/week | Sedentary control | 120 | ↓Tumor volume in the exercise group animals;  ↓Tumor number in the exercise group animals |
| Siewierska2018 | Female Sprague-Dawley rats (1-month-old) | Intraperitoneally injected with 180 mg/kg body weight of MNU. | Three-position treadmill (low, moderate, and high intensity training) | 13 weeks | 5 days/week | Sedentary control | 50 | ↑Ki-67 antigen expression in the exercise group animals; ↓Tumor volume in the exercise group animals. |
| Siewierska2020 | Female Sprague-Dawley rats (1-month-old) | Intraperitoneal injection of 180 mg/kg body weight of MNU. | Three-position treadmill (low, moderate and high intensity training) | 12 weeks | 5 days/week | Sedentary control | 50 | ↓Tumor volume in the exercise group animals; ↑Breast cancer cells apoptosis in the exercise group animals. |
| Smeda2017 | Female Balb/c mice | Injected orthotopically with 1 × 104 of 4T1 breast cancer cells. | Voluntary treadmill running | 5 weeks | NR | Sedentary control | 36 | ↑The number of secondary nodules formed in the lungs in the exercise group animals;  No modify the volume and size of primary tumor in the exercise group animals; |
| Steiner2013 | Female FVB/N and C3(1)/SV40Tag mice | Female FVB/N wild-type mice were bred with male heterozygous C3(1)/SV40Tag transgenic mice. Female offspring were genotyped using RT-PCR for the C3(1)/SV40Tag gene. | Voluntary treadmill running | 20 weeks | NR | Sedentary control | 27 | ↓Tumor growth in the exercise group animals;  ↓Tumor volume per tumor in the exercise group animals; ↑Tumor number in the exercise group animals; No inhibiting tumor initiation in the exercise group animals. |
| Thompson1992 | Female Sprague-Dawley rats (50 days old) | Intubated with 5 mg DMBA. | Treadmill running | 18 weeks | 5 days/week | Sedentary control | 138 | The tumorigenic response in exercised rats was enhanced. |
| Thompson1995 | Female F344 rats (50-57 days old) | Injected intraperitoneally with 50 mg MNU/kg body weight | Treadmill running | 12 weeks | 5 days/week | Sedentary control | 150 | ↓Tumor incidence in exercise group animals; ↓Tumor multiplicity in exercise group animals |
| Thompson199 | Female F344 rats (50 days old) | Intraperitoneal injection of the carcinogen MNU (35 mg/kg body weight) | Treadmill running | 26 weeks | 5 days/week | Sedentary control | 120 | ↓Tumor number in exercise group animals; ↓Tumor incidence in exercise group animals |
| Thompson2010 | Female Sprague-Dawley rats (21 days old) | Injected with 50 mg/kg MNU of body weight. | Treadmill running | NR | NR | Sedentary control | 110 | ↓Tumor number in exercise group animals;  ↓Tumor incidence in exercise group animals |
| Vulczak2020 | Female Balb/c mice (9 weeks old) | 4T1 cells (1 × 104) in suspension were injected orthotopically into the fourth-right dorsal mammary fat pad. | Treadmill running | 12 weeks | 5 days/week | Sedentary control | 36 | ↓Tumor volume in the exercise group animals; ↓Average tumor weight in the exercise group animals; ↓Tumor growth in the exercise group animals+K8:K11 |
| Wang2008 | Sprague Dawleyrats (4 weeks old) | Subcutaneously injected with DMBA 10mg /100g of body weight. | Swim training | 6 weeks | 4 days/week | Sham swimming | 60 | ↓The tumor-free time of mammary tumors in exercise group animals;  ↓Tumor number in exercise group animals; ↓Tumor incidence in exercise group animals |
| Welsch1995 | Female athymic nude mice(two-month-old) | Palpable MDA-MB231 human breast carcinomas cut into slices (2x4 mm, 0.1-0.3 mm thick), and subcutaneously implanted into recipient mice, three or four slices per mouse. | Treadmill running | 5 weeks | NR | Sedentary control | 10 | Growth of the human breast carcinomas was significantly inversely correlated with the mean number of miles that each mouse ran per day;  energy expenditure, via voluntary use of an activity wheel, can reduce significantly the growth of human breast carcinomas maintained in athymic nude mice. |
| Wen2010 | Female Balb/c mice | Mice inoculated with underarm BCAP-37 breast cancer cells. | Treadmill running | 4 weeks | 6 days/week | Sedentary control | 30 | ↓Tumor volume in exercise group animals |
| Wennerberg2020 | Female Balb/c mice (6-8 weeks old) | Mice were subcutaneously inoculated with 4T1 cells. | Treadmill running | 30 days | 5 days/week | Sedentary control | 12 | ↓Tumor progression in the exercise group animals; ↓Tumor volume in the exercise group animals |
| Westerlind2003 | Female Sprague-Dawley rats (20 days old) | Injected with MNU (50 mg/kg body weight). | Treadmill running | 6 weeks/8 weeks | 5 days/week | Sedentary control | 94 | ↓Tumor weight in exercise group animals;  ↓Tumor growth rate in exercise group animals |
| Woods1994 | Male CH/HeN mice (6 weeks old) | Inoculated subcutaneously with 2.5 x 105 mammary adenocarcinoma cells. | Treadmill running | 2 weeks | NR | Sedentary control | 88 | ↓Tumor weight in exercise group animals;  ↑Tumor incidence in exercise group animals |
| Zhu2008 | Female Sprague-Dawley rats | Injected with 50 mg 1-methyl-1-nitrosourea/kg body weight. | Voluntary treadmill running | 4 weeks | NR | Sedentary control | 120 | ↓Tumor number in exercise group animals; ↓Tumor incidence in exercise group animals; ↓Tumor multiplicity in exercise group animals; |
| Zhu2009 | Female Sprague-Dawley rats (3 weeks old) | Injected with 50 mg MNU/kg body weight. | Voluntary wheel running | NR | NR | Sedentary control | 54 | ↓Tumor incidence in the exercise group animals;  ↑Apoptosis in the exercise group animals |

**Abbreviations:** ↑ = increase; ↓ = decrease; DMBA = 7, 12-dimethylbenz(a)anthracene ; MNU = 1- methyl-1-nitrosourea; NR = no report

**References**

Abdalla, D.R., Murta, E.F., and Michelin, M.A. (2013). The influence of physical activity on the profile of immune response cells and cytokine synthesis in mice with experimental breast tumors induced by 7,12-dimethylbenzanthracene. *Eur J Cancer Prev* 22(3)**,** 251-258. doi: 10.1097/CEJ.0b013e3283592cbb.

Alizadeh, A.M., Heydari, Z., Rahimi, M., Bazgir, B., Shirvani, H., Alipour, S., et al. (2018). Oxytocin mediates the beneficial effects of the exercise training on breast cancer. *Experimental Physiology* 103(2)**,** 222-235. doi: 10.1113/EP086463.

Alvarado, A., Gil da Costa, R.M., Faustino-Rocha, A.I., Ferreira, R., Lopes, C., Oliveira, P.A., et al. (2017). Effects of exercise training on breast cancer metastasis in a rat model. *International Journal of Experimental Pathology* 98(1)**,** 40-46. doi: 10.1111/iep.12225.

Amani Shalamzari, S., Agha-Alinejad, H., Alizadeh, S., Shahbazi, S., Kashani Khatib, Z., Kazemi, A., et al. (2014). The effect of exercise training on the level of tissue IL-6 and vascular endothelial growth factor in breast cancer bearing mice. *Iranian Journal of Basic Medical Sciences* 17(4)**,** 231-236.

Aveseh, M., Nikooie, R., and Aminaie, M. (2015). Exercise-induced changes in tumour LDH-B and MCT1 expression are modulated by oestrogen-related receptor alpha in breast cancer-bearing BALB/c mice. *J Physiol* 593(12)**,** 2635-2648. doi: 10.1113/jp270463.

Bianco, T.M., Abdalla, D.R., Desidério, C.S., Thys, S., Simoens, C., Bogers, J.P., et al. (2017). The influence of physical activity in the anti-tumor immune response in experimental breast tumor. *Immunol Lett* 190**,** 148-158. doi: 10.1016/j.imlet.2017.08.007.

Buss, L.A., Ang, A.D., Hock, B., Robinson, B.A., Currie, M.J., and Dachs, G.U. (2020). Effect of post-implant exercise on tumour growth rate, perfusion and hypoxia in mice. *PLoS One* 15(3)**,** e0229290. doi: 10.1371/journal.pone.0229290.

Buss, L.A., and Dachs, G.U. (2018). Voluntary exercise slows breast tumor establishment and reduces tumor hypoxia in ApoE(-/-) mice. *J Appl Physiol (1985)* 124(4)**,** 938-949. doi: 10.1152/japplphysiol.00738.2017.

Cohen, L.A., Kendall, M.E., Meschter, C., Epstein, M.A., Reinhardt, J., and Zang, E. (1993). Inhibition of rat mammary tumorigenesis by voluntary exercise. *In Vivo* 7(2)**,** 151-158.

Colbert, L.H., Westerlind, K.C., Perkins, S.N., Haines, D.C., Berrigan, D., Donehower, L.A., et al. (2009). Exercise effects on tumorigenesis in a p53-deficient mouse model of breast cancer. *Med Sci Sports Exerc* 41(8)**,** 1597-1605. doi: 10.1249/MSS.0b013e31819f1f05.

Cui Mu (2017). Effects of aerobic exercise on cancer-induced fatigue and tumor-bearing growth in SD rats with breast cancer

. *Chinese Journal of Gerontology* 37(18).

da Costa, T. S. R., Urias, U., Negrao, M. V., Jordão, C. P., Passos, C. S., Gomes- Santos, I. L., et al. (2021). Breast Cancer Promotes Cardiac Dysfunction through Deregulation of Cardiomyocyte Ca 2+ -Handling Protein Expression that Is Not Reversed by Exercise Training. J Am Heart Assoc. 10 (5), e018076. doi:10.1161/ jaha.120.018076

Faustino-Rocha, A.I., Gama, A., Oliveira, P.A., Alvarado, A., Neuparth, M.J., Ferreira, R., et al. (2017). Effects of lifelong exercise training on mammary tumorigenesis induced by MNU in female Sprague–Dawley rats. *Clinical and Experimental Medicine* 17(2)**,** 151-160. doi: 10.1007/s10238-016-0419-0.

Faustino-Rocha, A.I., Silva, A., Gabriel, J., Gil da Costa, R.M., Moutinho, M., Oliveira, P.A., et al. (2016). Long-term exercise training as a modulator of mammary cancer vascularization. *Biomed Pharmacother* 81**,** 273-280. doi: 10.1016/j.biopha.2016.04.030.

Figueira, M.C., Silva, C., Padrão, A., Oliveira, P.A., Ferreira, R.P., and Duarte, J.A. (2018). Exercise Training-Induced Modulation in Microenvironment of Rat Mammary Neoplasms. *International Journal of Sports Medicine*.

Garritson, J., Haughian, J., Pullen, N., and Hayward, R. (2019). Exercise Reduces Proportions of Tumor Resident Myeloid‐Derived Suppressor Cells. *The FASEB Journal* 33(S1).

Gholamian, S., Hosseini, S.R.A., Rashidlamir, A., and Aghaalinejad, H. (2020). The effects of interval aerobic training on mesenchymal biomarker gene expression, the rate of tumor volume, and cachexia in mice with breast cancer. *Iranian Journal of Basic Medical Sciences* 23(2)**,** 244-250. doi: 10.22038/IJBMS.2019.39535.9375.

Goh, J., Tsai, J., Bammler, T.K., Farin, F.M., Endicott, E., and Ladiges, W.C. (2013). Exercise training in transgenic mice is associated with attenuation of early breast cancer growth in a dose-dependent manner. *PLoS One* 8(11)**,** e80123. doi: 10.1371/journal.pone.0080123.

Isanejad, A., Alizadeh, A.M., Amani Shalamzari, S., Khodayari, H., Khodayari, S., Khori, V., et al. (2016). MicroRNA-206, let-7a and microRNA-21 pathways involved in the anti-angiogenesis effects of the interval exercise training and hormone therapy in breast cancer. *Life Sciences* 151**,** 30-40. doi: 10.1016/j.lfs.2016.02.090.

Jones, L.W., Eves, N.D., Courneya, K.S., Chiu, B.K., Baracos, V.E., Hanson, J., et al. (2005). Effects of exercise training on antitumor efficacy of doxorubicin in MDA-MB-231 breast cancer xenografts. *Clin Cancer Res* 11(18)**,** 6695-6698. doi: 10.1158/1078-0432.Ccr-05-0844.

Jones, L.W., Viglianti, B.L., Tashjian, J.A., Kothadia, S.M., Keir, S.T., Freedland, S.J., et al. (2010). Effect of aerobic exercise on tumor physiology in an animal model of human breast cancer. *J Appl Physiol (1985)* 108(2)**,** 343-348. doi: 10.1152/japplphysiol.00424.2009.

Leila, A., Reza, K.M., Abbasali, G., Reza, M., and Zahra, M. (2015). Effects of exercise training on development of breast cancer in mice. *Biomedical and Pharmacology Journal* 8(2)**,** 785-792. doi: 10.13005/bpj/827.

Lyv Di, S.Y., Li Xiaobiao, Chen Yanan, Wu Yinyu, Li Jingwen, (2021). Efects of Moderate Intensity Aerobic Exercise on Tumor Growth in 4T1Breast Cancer Mice by nhibiting Excesively Activated NF-κB Pathway. *Journal of Southwest China Normal University(Natural Science Edition)* 46(4)**,** 53-60. doi: 10.13718/j.cnki.xsxb.2021.04.011.

Malicka, I., Siewierska, K., Pula, B., Kobierzycki, C., Haus, D., Paslawska, U., et al. (2015). The effect of physical training on the N-methyl-N-nitrosourea-induced mammary carcinogenesis of Sprague–Dawley rats. *Experimental Biology and Medicine* 240(11)**,** 1408-1415. doi: 10.1177/1535370215587532.

Mann, P.B., Jiang, W., Zhu, Z., Wolfe, P., McTiernan, A., and Thompson, H.J. (2010). Wheel running, skeletal muscle aerobic capacity and 1-methyl-1-nitrosourea induced mammary carcinogenesis in the rat. *Carcinogenesis* 31(7)**,** 1279-1283. doi: 10.1093/carcin/bgq063.

Moore, C., and Tittle, P.W. (1973). Muscle activity, body fat, and induced rat mammary tumors. *Surgery* 73(3)**,** 329-332.

Murphy, E.A., Davis, J.M., Barrilleaux, T.L., McClellan, J.L., Steiner, J.L., Carmichael, M.D., et al. (2011). Benefits of exercise training on breast cancer progression and inflammation in C3(1)SV40Tag mice. *Cytokine* 55(2)**,** 274-279. doi: 10.1016/j.cyto.2011.04.007.

Nasiri, M., Peeri, M., and Matinhomaei, H. (2017). Endurance training attenuates angiogenesis following breast cancer by regulation of MiR-126 and MiR-296 in breast cancer bearing mice. *International Journal of Cancer Management* 10(6). doi: 10.5812/ijcm.8067.

Pu Jinsong, Wang Yu, Liu Yanna, and Yang Tianfu (2008). Effects of exercise on MNU-induced breast cancer in rats. *Journal of Sichuan University (Medical Science edition)* 39(2)**,** 320-322.

Qi, D.J., Zhang, Q.F., Feng, L., and Liu, B. (2013). Effect of aerobic exercise on 7, 12-dimethylbenz{a}anthracene induced mammary cancer in rats. *Chinese Journal of Cancer Prevention and Treatment* 20(24)**,** 1878-1881.

Siewierska, K., Malicka, I., Kobierzycki, C., Grzegrzolka, J., Piotrowska, A., Paslawska, U., et al. (2020). Effect of physical training on the levels of sex hormones and the expression of their receptors in rats with induced mammary cancer in secondary prevention model – Preliminary study. *In Vivo* 34(2)**,** 495-501. doi: 10.21873/invivo.11800.

Siewierska, K., Malicka, I., Kobierzycki, C., Paslawska, U., Cegielski, M., Grzegrzolka, J., et al. (2018). The impact of exercise training on breast cancer. *In Vivo* 32(2)**,** 249-254. doi: 10.21873/invivo.11231.

Smeda, M., Przyborowski, K., Proniewski, B., Zakrzewska, A., Kaczor, D., Stojak, M., et al. (2017). Breast cancer pulmonary metastasis is increased in mice undertaking spontaneous physical training in the running wheel; a call for revising beneficial effects of exercise on cancer progression. *American Journal of Cancer Research* 7(9)**,** 1926-1936.

Steiner, J.L., Davis, J.M., McClellan, J.L., Enos, R.T., and Murphy, E.A. (2013). Effects of voluntary exercise on tumorigenesis in the C3(1)/SV40Tag transgenic mouse model of breast cancer. *International Journal of Oncology* 42(4)**,** 1466-1472. doi: 10.3892/ijo.2013.1827.

Thompson, H.J. (1992). Effect of amount and type of exercise on experimentally induced breast cancer. *Advances in Experimental Medicine and Biology* 322**,** 61-71.

Thompson, H.J., Westerlind, K.C., Snedden, J., Briggs, S., and Singh, M. (1995a). Exercise intensity dependent inhibition of 1-methyl-1-nitrosourea induced mammary carcinogenesis in female F-344 rats. *Carcinogenesis* 16(8)**,** 1783-1786. doi: 10.1093/carcin/16.8.1783.

Thompson, H.J., Westerlind, K.C., Snedden, J.R., Briggs, S., and Singh, M. (1995b). Inhibition of mammary carcinogenesis by treadmill exercise. *J Natl Cancer Inst* 87(6)**,** 453-455. doi: 10.1093/jnci/87.6.453.

Thompson, H.J., Wolfe, P., McTiernan, A., Jiang, W., and Zhu, Z. (2010). Wheel running-induced changes in plasma biomarkers and carcinogenic response in the 1-methyl-1-nitrosourea-induced rat model for breast cancer. *Cancer Prev Res (Phila)* 3(11)**,** 1484-1492. doi: 10.1158/1940-6207.Capr-10-0078.

Vulczak, A., Souza, A.O., Ferrari, G.D., Azzolini, A.E.C.S., Pereira-Da-silva, G., and Alberici, L.C. (2020). Moderate exercise modulates tumor metabolism of triple-negative breast cancer. *Cells* 9(3). doi: 10.3390/cells9030628.
