# Supplementary material for: Effect of Exercise on Breast Cancer: A Systematic Review and Meta-analysis of Animal Experiments
Source: Front Mol Biosci. 2022 Jun 6;9:843810. doi: 10.3389/fmolb.2022.843810 (PMC9208379; doi:10.3389/fmolb.2022.843810)
Supplement: Supplementary file 2 [file Table2.docx]

**Supplementary file 2. Search strategy**

| **Set** | **Query** |
| --- | --- |
| **#1** | (("Breast Neoplasms"[Mesh] OR "Breast Cancer Lymphedema"[Mesh]) AND (("Exercise"[Mesh]) OR "Sports"[Mesh])) AND (("Models, Animal"[Mesh]) OR "Animal Experimentation"[Mesh]) |
| **#2** | ("Models, Animal"[Mesh]) OR "Animal Experimentation"[Mesh] |
| **#3** | ("Exercise"[Mesh]) OR "Sports"[Mesh] |
| **#4** | (#3) AND (#2) AND #1 |
