# Supplementary material for: Effect of Exercise on Breast Cancer: A Systematic Review and Meta-analysis of Animal Experiments
Source: Front Mol Biosci. 2022 Jun 6;9:843810. doi: 10.3389/fmolb.2022.843810 (PMC9208379; doi:10.3389/fmolb.2022.843810)
Supplement: Supplementary file 3 [file Table3.DOCX]

**Supplementary file 3. The excluded studies with the reasons for exclusion**

| **Study/year** | **Title** | **Reason for exclusion** |
| --- | --- | --- |
| Agha-Alinejad2015 | Effect of 6 weeks endurance training on serum tnf-α in breast cancer bearing mice | 4 |
| Amani2018 | The effect of aerobic training on tumor growth, adiponectin, leptin and ghrelin in mice with breast cancer | 4 |
| Chekachak2017 | Assessment of aerobic training with selenium nanoparticles supplementation effects on cytokines levels of liver tissue in 4T1 breast cancer mice | 4 |
| Cohen1988 | Influence of dietary fat, caloric restriction, and voluntary exercise on N-nitrosomethylurea-induced mammary tumorigenesis in rats | 1 |
| Dewhirst2016 | Exercise and cancer progression Preclinical evidence | 3 |
| Esmailiyan2021 | Comparing the effects of 4 and 12 weeks of aerobic training on tumor volume in female mice with breast cancer | 4 |
| Gillette1997 | Energy availability and mammary carcinogenesis: Effects of calorie restriction and exercise | 1 |
| Gomes2020 | Age-induced accumulation of methylmalonic acid promotes tumour progression | 2 |
| Hoffman-Goetz1994 | Exercise and breast cancer: review and critical analysis of the literature | 2 |
| Hoffman-Goetz2003 | Physical activity and cancer prevention: animal-tumor models | 2 |
| Igor2021 | Exercise Training Improves Tumor Control by Increasing CD8 T-cell Infiltration via CXCR3 Signaling and Sensitizes Breast Cancer to Immune Checkpoint Blockade | 3 |
| Khalighfard2018 | The effect of 8 weeks of interval aerobic exercise before and after induction of breast cancer on serum level of irisin and tumor growth in balb/c mice | 4 |
| Khori2015 | Effects of exercise training together with tamoxifen in reducing mammary tumor burden in mice: Possible underlying pathway of MIR-21 | 1 |
| Kim2019 | Steady low intensity physical activity and healthy dietary habits are differently affect breast cancer progression | 1 |
| Lee2015 | Impact of aerobic exercise on tumor oxygenation and perfusion in breast cancer | 3 |
| Li2016 | Can Exercise Ameliorate Aromatase Inhibitor-Induced Cognitive Decline in Breast Cancer Patients? | 2 |
| Lønbro2018 | High intensity treadmill running reduces tumour hypoxia in mice | 3 |
| Moghadam2017 | The protective effect of aerobic exercise on breast cancer by TGFβ protein and Smad and MMP2 gene in femal mice | 4 |
| Molanouri2017 | Combined effect of aerobic interval training and selenium nanoparticles on expression of IL-15 and IL-10/TNF-α ratio in skeletal muscle of 4T1 breast cancer mice with cachexia | 1 |
| Mohammad2021 | Gene expression of angiogenesis and apoptotic factors in female BALB/c mice with breast cancer after eight weeks of aerobic training | 3 |
| NA2020 | 2020 Annual Meeting of the American Society for Bone and Mineral Research | 3 |
| Nelson2018 | The impact of aerobic exercise on breast cancer progression | 3 |
| Padovani2009 | Distinct effects of calorie restriction and exercise on mammary gland gene expression in C57BL/6 mice | 1 |
| Shahvali2021 | The effect of aerobic training on tumor growth and expression of Bcl-2 gene and protein in female mice with breast cancer | 4 |
| Shirali2017 | Effects of Six Weeks Endurance Training and Aloe Vera Supplementation on COX-2 and VEGF Levels in Mice with Breast Cancer | 1 |
| Shiri2014 | Effect of six weeks endurance training on tumor tissue IL-10 cytokine levels in breast cancer bearing mice | 4 |
| Thompson1989 | Effect of type and amount of dietary fat on the enhancement of rat mammary tumorigenesis by exercise | 1 |
| Thompson2008 | Evaluation of the current knowledge limitations in breast cancer research: a gap analysis | 2 |
| Viguera2003 | Influence of exercise, oestrogen therapy and a diet high ω-3 fatty acids on the lipid composition of the mammary tissue, in the Wistar rat | 2 |
| Wakefield2020 | Aerobic exercise delays intraductal breast cancer growth and suppresses doxorubicin-induced skeletal and cardiac muscle toxicities | 3 |
| Wiggins2016 | The impact of aerobic exercise on the tumor microenvironment | 2 |
| Yazdanshenas2020 | Effect of 10 weeks of high-intensity interval training on protein levels of nf-kb and expression of atrogin-1 and murf-1 in cardiomyocytes of female mice with breast cancer | 4 |

Reason 1: exercise combined with calorie restriction

Reason 2: review or other analysis

Reason 3: conference article or no data in tumor outcomes

Reason 4: language other than Chinese and English
