# Supplementary material for: Effect of Exercise on Breast Cancer: A Systematic Review and Meta-analysis of Animal Experiments
Source: Front Mol Biosci. 2022 Jun 6;9:843810. doi: 10.3389/fmolb.2022.843810 (PMC9208379; doi:10.3389/fmolb.2022.843810)

**Supplementary file 4. Sensitivity analyses of outcomes**

**Tumor weight**


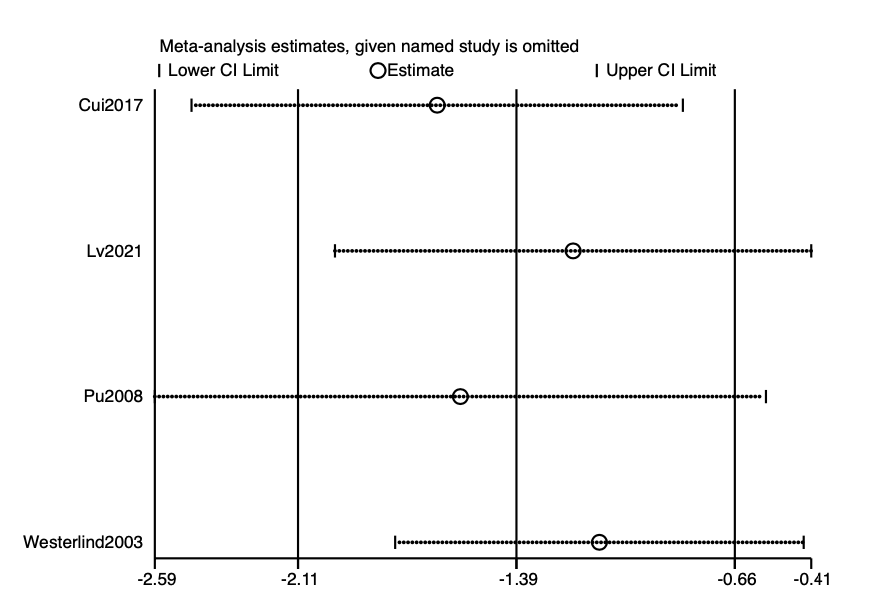


**Tumor number**


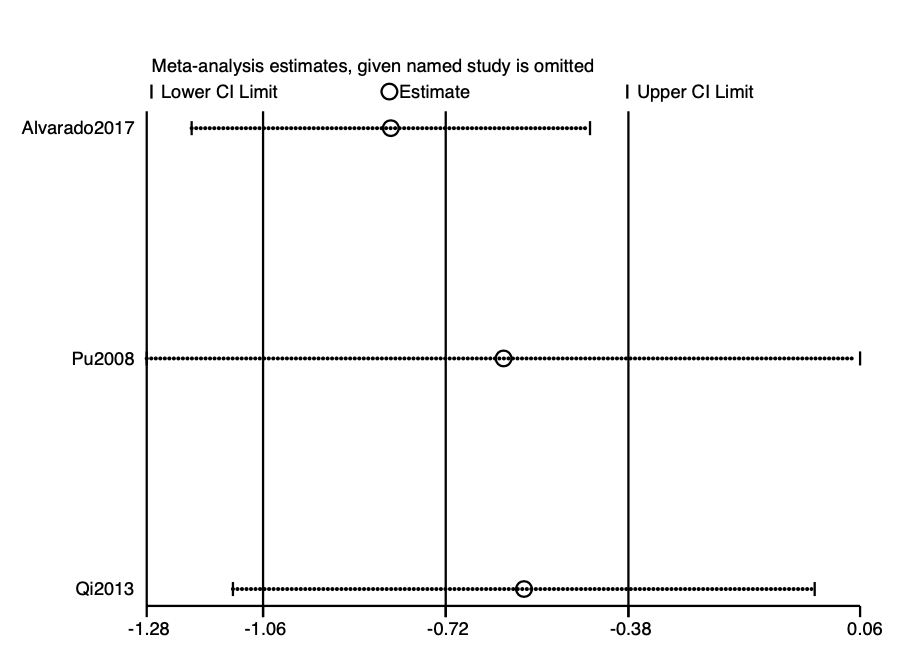


**Tumor incidence (motorized wheel/high-intensity)**


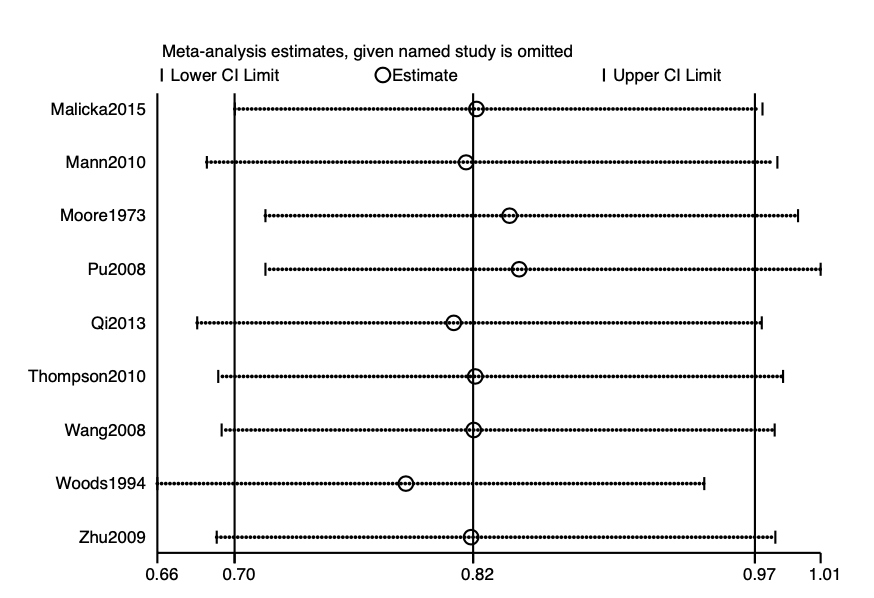


**Tumor incidence (ree wheel/low-intensity)**


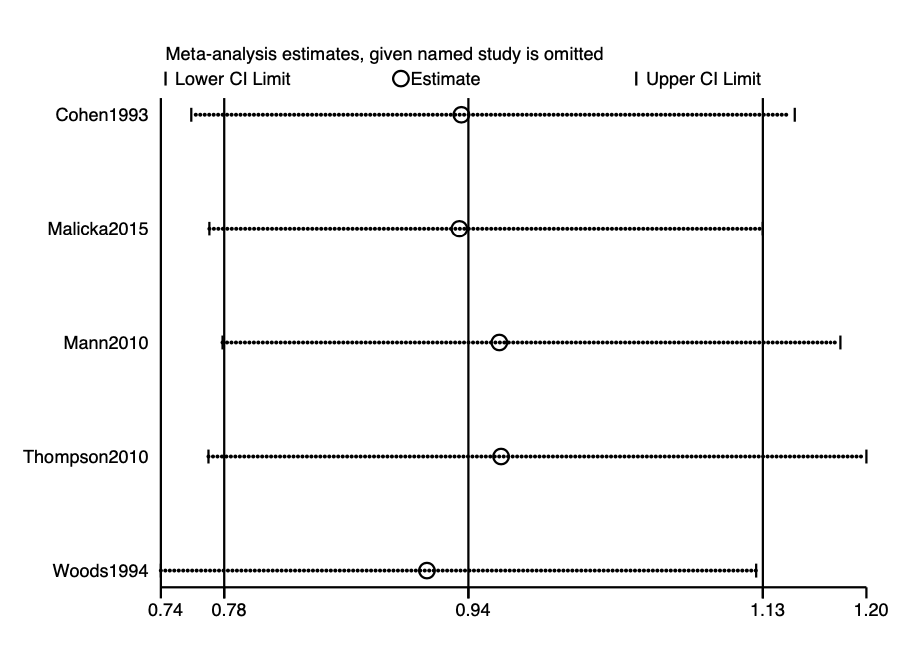

Supplement: Supplementary file 4 [file Table4.DOCX]
